# Psychological comorbidities in osteoarthritis in Germany

**DOI:** 10.1038/s41598-023-29867-4

**Published:** 2023-02-18

**Authors:** Nike Walter, Thilo Hinterberger, Dominik Szymski, Volker Alt, Markus Rupp

**Affiliations:** 1 Department for Trauma Surgery, University Hospital Regensburg, Franz-Josef-Strauß-Allee 11, 93053 Regensburg, Germany; 2 Department for Psychosomatic Medicine, University Hospital Regensburg, Regensburg, Germany

**Keywords:** Epidemiology, Rheumatology

## Abstract

Osteoarthritis is a degenerative joint disease associated with pain, loss of function and reduced quality of life. Concomitant psychological disorders can significantly influence treatment outcomes. Therefore, we aimed to answer the following research questions: (1) How has the incidence of primary coxarthrosis and gonarthrosis developed over the last decade? (2) How high is the prevalence of osteoarthritis patients with concomitant psychological diagnoses? (3) Which psychological comorbidities are most prevalent in coxarthrosis and gonarthrosis patients, respectively? For this cross-sectional study, a dataset provided by the Federal Statistical Office (Destatis) consisting of annual, Germany-wide ICD-10 diagnosis codes from 2009 to 2019 was analysed. Incidences of the codes “M16.1” and “M17.1”, unilateral primary coxarthrosis and unilateral primary gonarthrosis, were quantified. Prevalence rates of secondary diagnoses of the chapter F of the ICD-10 were determined. Incidences were 230.7/100,000 inhabitants for coxarthrosis and 224.2/100,000 inhabitants for gonarthrosis. Patients with psychological comorbidities constituted 9.0% of coxarthrosis cases and 8.9% of gonarthrosis cases, respectively. Between 2009 through 2019, the proportion of patients with a concomitant “F” diagnoses of the ICD-10 increased by + 37.8% for coxarthrosis and by 17.9% for gonarthrosis. The most prevalent secondary diagnoses were affective disorders (F3), whereby numbers increased over the years. Increasing psychological comorbidities advocate for the implementation of screening tools, prevention strategies, interdisciplinary approaches and psychological support in the treatment of osteoarthritis.

## Introduction

Osteoarthritis (OA) represents a degenerative joint disease affecting about 3.3 to 3.6% of the population worldwide^1^. OA is associated with pain, loss of function and significantly reduced quality of life^2^. In 2010, OA was ranked as the 11th highest contributor to global disability in 2010^3^. According to guidelines, therapy recommendations include pharmacological treatments such as the prescription of paracetamol, NSAIDs, corticosteroid injections and tramadol^4^. However, recent reviews and meta-analyses showed that the benefits are limited^5–7^. Therefore, for end stage OA, joint replacement as a life-enhancing procedure is often the therapy choice. In Germany, primary total knee or hip arthroplasty is among the most common procedures, with an increase in the number of surgeries of up to 45% predicted for the year 2040^8^. It was estimated that 80% of total hip arthroplasty (THA) and 96% of total knee arthroplasty (TKA) surgeries are due to OA^9^.

It is well established that somatic comorbidities influence OA treatment outcomes. For instance, the number of somatic comorbidities such as hypertension and heart diseases were associated with worse quality of life, more problems with walking, higher pain severity and a higher amount of pain medication^10^. Also, respiratory, cardiovascular diseases, type 2 diabetes mellitus and obesity were shown to negatively impact OA patients’ physical activity^11^. Besides, psychological disorders also play an essential role. For instance, the concomitant diagnosis of depression leads to increased risk of infection after joint replacement and is associated with worse clinical outcomes^12–14^. It is known that patients with OA are at higher risks of developing psychological symptoms^15^, however, the prevalence of psychological comorbidities in OA based on nationwide registry data has not been analyzed. To estimate future demands and foresee developments, which could be influenced by a more comprehensive biopsychosocial approach to orthopedic care^16^, a determination of the recent trends of the nationwide burden of psychological comorbidities in OA is required.

Therefore, we aimed to answer the following research questions: (1) How has the incidence of primary coxarthrosis and gonarthrosis developed over the last decade? (2) How high is the prevalence of osteoarthritis patients with concomitant psychological diagnoses? (3) Which psychological comorbidities are most prevalent in knee and hip osteoarthritis patients, respectively?

## Material and methods

For this cross-sectional study, data consisting of annual ICD-10 diagnosis codes from German medical institutions between 2009 through 2019 was provided by the Federal Statistical Office of Germany (Destatis). These include all inpatient diagnosis, which are reported from medical institutions of all 16 German federal states. The ICD-10 codes “M16.1” and “M17.1” were used to identify patients aged 20 years or older diagnosed with unilateral primary coxarthrosis and unilateral primary gonarthrosis, respectively (Table 1). Incidences were calculated based on Germany’s historical population aged 20 years or older provided by Destatis. Here, the number of inhabitants in each of the 16 German federal states was considered by year of birth for each year of the period 2009 through 2019. The deadline of each year was December 31. Incidence rate ratios (IRR) with the corresponding 95% confidence interval (CI) and percentage changes were calculated by dividing the incidence in 2019 by the incidence of the year 2009. Further, the number of inpatients with the primary diagnoses “M16.1” and “M17.1” and a secondary diagnosis of the chapter F (F0-F9) of the ICD-10, mental and behavioral disorders was estimated. The distribution of secondary diagnoses was examined for the diagnoses “F0, F1, F2, F3, F4, F5, F6, F7, F8” and “F9”, respectively, as described in a previous study (Table 1)^17^. Data was analysed using SPSS statistics version 24.0 (IBM, SPSS Inc., Armonk, NY). Descriptive statistics were calculated for all variables. Incidence rates were compared using the two-sample z-test. Significance was set at* p* < 0.05.

**Table 1:** Used ICD-10 codes with descriptions and examples of the disorder.

ICD-10 code	Description
M16.1	Primary coxarthrosis; unilateral
M17.1	Primary gonarthrosis; unilateral
F0	Organic, including symptomatic, mental disorders
F1	Mental and behavioural disorders due to psychoactive substance use
F2	Schizophrenia, schizotypal and delusional disorders
F3	Mood [affective] disorders
F4	Neurotic, stress-related and somatoform disorders
F5	Behavioural syndromes associated with psychological disturbances and physical factors
F6	Disorders of adult personality and behaviour
F7	Mental retardation
F8	Disorders of psychological development
F9	Behavioural and emotional disorders with onset usually occurring in childhood and adolescence

This is a purely observational study. The Research Ethics Committee of the University Hospital Regensburg has confirmed that no approval and no informed consent is required. The study was performed in accordance with the ethical standards in the 1964 Declaration of Helsinki. No administrative permission was required to access the analysed dataset. The data was anonymised before its use.

### Ethics approval and consent to participate

The Research Ethics Committee of the University Hospital Regensburg has confirmed that no approval and no informed consent is required.

## Results

**Figure 1:**
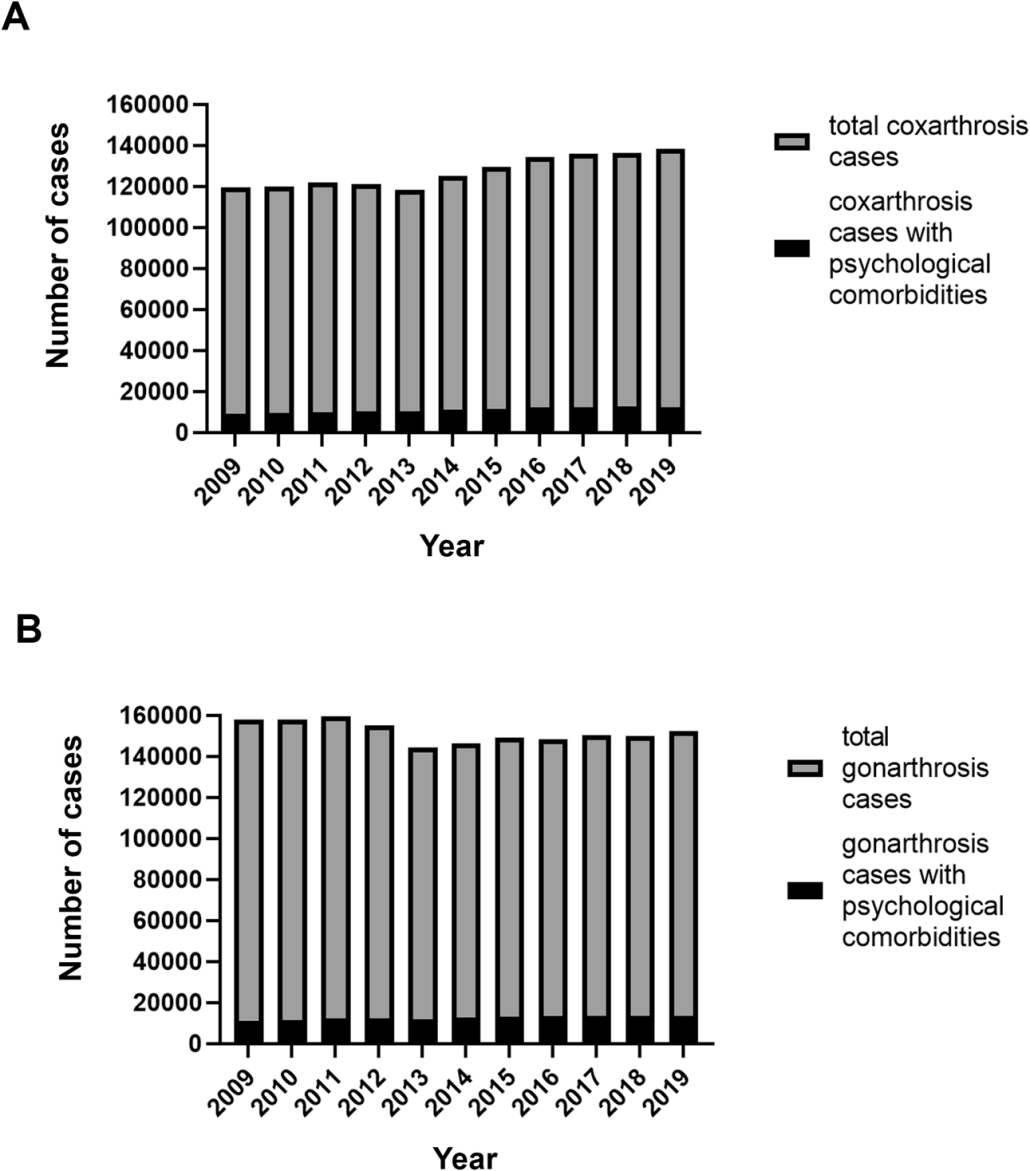
Historical development of (**A**) total coxarthrosis cases shown in grey and coxarthrosis cases with psychological comorbidities shown in black, (**B**) total gonarthrosis cases shown in grey and gonarthrosis cases with psychological comorbidities shown in black from 2009 through 2019 in Germany.

**Figure 2:**
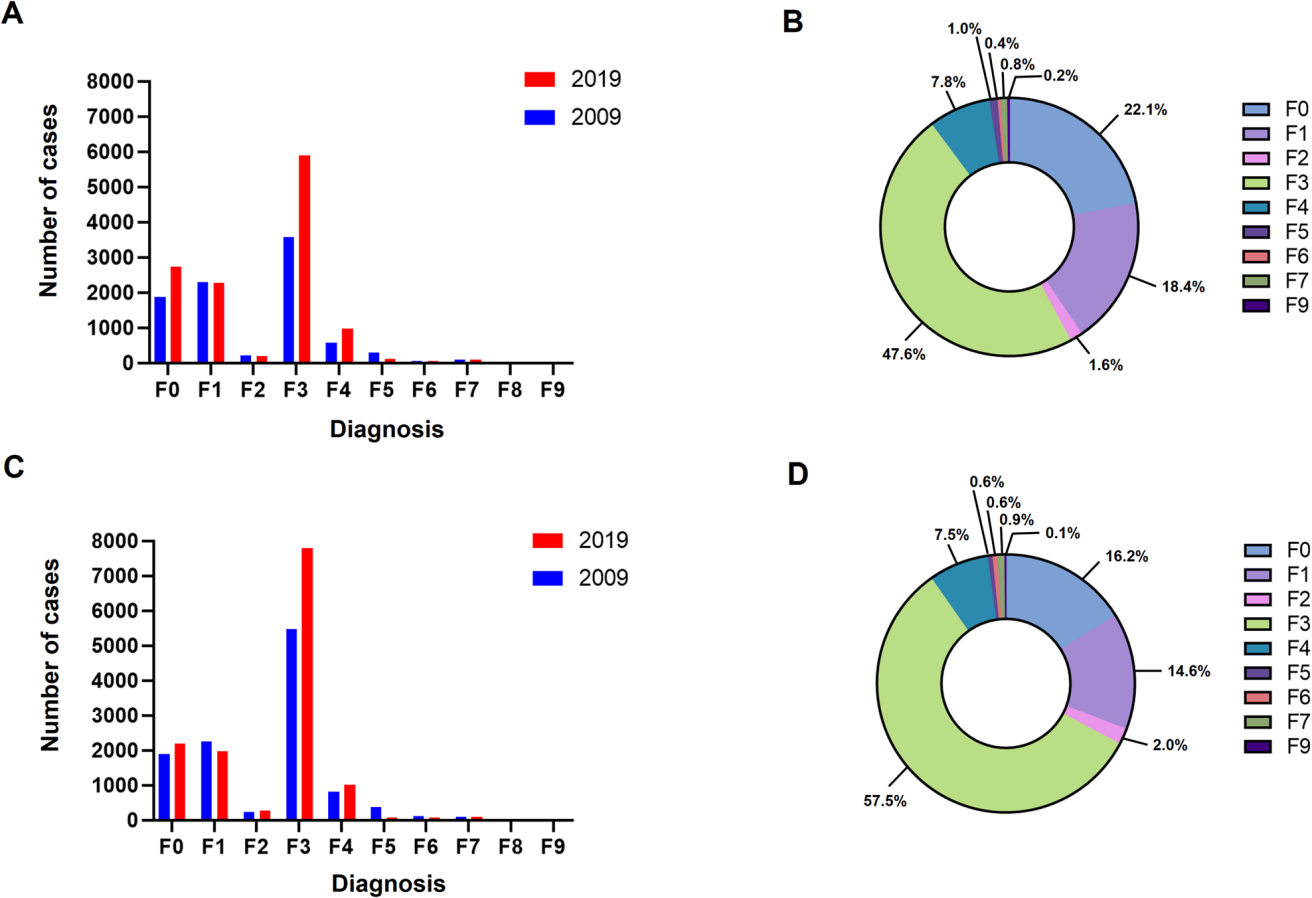
(**A**) Comparison of psychological comorbidities according to chapter F of the ICD-10 among coxarthrosis patients in the year 2009 shown in blue and 2019 shown in red. (**B**) Distribution of the ICD-10 chapter F diagnoses in coxarthrosis patients in 2019 visualized as percentages. (**C**) Comparison of psychological comorbidities according to chapter F of the ICD-10 among gonarthrosis patients in the year 2009 shown in blue and 2019 shown in red. (**D**) Distribution of the ICD-10 chapter F diagnoses in gonarthrosis patients in 2019 visualized as percentages.

In 2019, a total number of 138,232 coxarthrosis cases was registered in Germany. In comparison to 119,542 cases in 2009, the incidence increased by 13.1% to 203.7 cases per 100,000 inhabitants (IRR: 1.13, 95%CI 0.93–1.38, p = 0.114). Out of these, 9.0% were diagnosed with psychological comorbidities according to chapter F (12,389 cases) (Fig. 1). The percentage of patients with concomitant diagnoses steadily increased over the last decade by + 37.8% (IRR: 1.19, 95%CI 1.16–1.23, p < 0.001) (Table 2). The largest proportion of coxarthrosis patients with psychological comorbidities was diagnosed with a F3 ICD-10 code, affective disorders (47.6%) in 2019. This was followed by diagnoses of F0, organic, including symptomatic, mental disorders with 22.1% and F1, mental and behavioral disorders due to psychoactive substance use with 18.4% (Fig. 2). Compared to 2009, the highest increases were found for F4, neurotic, stress-related and somatoform disorders (+ 70.6%), F3, affective disorders (+ 65.3%) and F0 diagnoses (+ 45.6%). The highest decrease was found for F5, behavioural syndromes associated with psychological disturbances and physical factors (− 58.6%) and F6, disorders of adult personality and behaviour (− 11.8%) (Table 4).

**Table 2:** Historical development of total coxarthrosis cases and cases with psychological comorbidities from 2009 through 2019 in Germany.

Year	Coxarthrosis total	Incidence per 100,000 inhabitants	Psychological comorbidities				
			Total	Percentage	Relative to 2009	IRR relative to the preceding year	p-value derived from two-sample z-test
2009	119,542	180.0	8990	7.5			
2010	120,059	180.4	9540	8.0	+ 13.3%	1.06	< 0.001
2011	122,103	186.7	10,044	8.2	+ 19.2%	1.04	0.028
2012	121,148	184.5	10,378	8.6	+ 23.2%	1.04	0.009
2013	118,295	179.4	10,251	8.7	+ 21.7%	1.01	0.450
2014	125,090	187.6	11,132	8.9	+ 32.2%	1.03	0.078
2015	129,688	193.3	11,629	9.0	+ 38.1%	1.01	0.612
2016	134,283	199.1	12,414	9.2	+ 47.4%	1.03	0.040
2017	135,776	201.0	12,155	9.0	+ 44.3%	0.97	0.030
2018	136,395	201.4	12,649	9.3	+ 50.2%	1.04	0.017
2019	138,232	203.7	12,389	9.0	+ 37.8%	0.97	0.021

The number of gonarthrosis patients slightly fluctuated over the years with an overall decrease of − 5.7% between 2009 through 2019 reaching an incidence of 224.2 cases per 100,000 inhabitants with 152,160 cases in total in 2019 (IIR: 0.94, 95%CI 0.79–1.13, p = 0.265). In 2019, 8.9% of all gonarthrosis patients were diagnosed with a concomitant psychological disorder (13,557 cases) (Fig. 1). Compared to 2009, the percentage of patients with psychological comorbidities increased statistically significant by + 17.9% (IRR: 1.24, 95%CI 1.21–1.28, p < 0.001) (Table 3). Psychological disorders mainly constituted F3, affective disorders, diagnoses with 57.5% (Fig. 2). In comparison to 2009, affective disorders (F3) showed the highest increase (+ 42.0%), followed by F4, neurotic, stress-related and somatoform disorders (+ 25.6%). The highest decrease was also identified for F5, behavioural syndromes associated with psychological disturbances and physical factors (− 77.4%), followed by F6, disorders of adult personality and behaviour (− 23.2%). In addition, the diagnosis mental and behavioural disorders due to psychoactive substance use (F1) was lowered (− 12.3) (Table 4).

**Table 3:** Historical development of total gonarthrosis cases and cases with psychological comorbidities from 2009 through 2019.

Year	Gonarthrosis total	Incidence per 100,000 inhabitants	Psychological comorbidities				
			Total	Percentage	Relative to 2009	IRR relative to the preceding year	p-value derived from two-sample z-test
2009	157,846	237.7	11,303	7.2			
2010	157,827	237.2	11,720	7.4	+ 11.25%	1.04	0.028
2011	159,407	243.7	12,241	7.7	+ 16.19%	1.03	0.039
2012	155,172	236.3	12,387	8.0	+ 17.58%	1.04	0.015
2013	144,144	218.6	12,106	8.4	+ 14.91%	1.05	0.001
2014	146,533	219.8	12,691	8.7	+ 20.47%	1.03	0.046
2015	149,123	222.2	13,083	8.8	+ 24.19%	1.01	0.394
2016	148,521	220.2	13,622	9.2	+ 29.30%	1.05	0.003
2017	150,172	222.3	13,446	9.0	+ 27.63%	0.98	0.105
2018	149,999	221.5	13,406	8.9	+ 27.25%	1.00	0.903
2019	152,160	224.2	13,557	8.9	+ 17.9%	1.00	0.836

**Table 4:** Distribution of psychological comorbidities according to the Chapter F of the ICD-10 in 2019.

ICD-10 diagnosis	Coxarthrosis [%]	Difference to 2009 [%]	Gonarthrosis [%]	Difference to 2009 [%]
F0	22.1	+ 45.6	16.2	+ 16.2
F1	18.4	− 0.31	14.6	− 12.3
F2	1.6	− 6.67	2.0	+ 14.6
F3	47.6	+ 65.3	57.5	+ 42.0
F4	7.8	+ 70.6	7.5	+ 25.6
F5	1.0	− 58.6	0.6	− 77.4
F6	0.4	− 11.3	0.6	− 23.2
F7	0.8	+ 4.3	0.90	− 4.0
F8	0.00	0.0	0.0	0.0
F9	0.2	+ 11.8	0.1	− 26.9

## Discussion

In this nationwide analysis, trends in the epidemiology of arthrosis cases with psychological comorbidities were determined. Between 2009 through 2019, the percentage of OA patients with a concomitant diagnoses of a psychological disorder increased, yielding a share of 9.0% of all coxarthrosis patients and 8.9% of gonarthrosis patientsin 2019. The majority of patients with OA suffered from affective disorders (F3).

Here, the highest increase over the considered time period was found for affective disorders, including depression, in patients with gonarthrosis as well as coxarthrosis. This might be explainable by the fact, that OA is associated with pain being a primary symptom of the disease, which is frequently persistent and chronic^18,19^. Multiple studies have shown that chronic pain depicts a fundamental risk factor for the development of depression and the intertwined combination of both is commonly encountered in clinical settings^20^. In addition, the results showed an increase in F4 diagnoses, especially in the coxarthrosis cohort. Amongst other, the chapter F4 covers symptoms of anxiety and fear. It is known, that anxiety is common in patients with long-term conditions, although not adequately addressed in management guidelines^21^. Also here, a reciprocal relationship with chronic pain has been described, and anxiety has been found to be related to poorer physical function in OA patients^22^. However, whether the concomitant diagnoses are a consequence of OA remains speculative. Further, the increasing prevalence may be influenced by the fact, that even though the stigmatization of mental disorders remains an issue, mental health problems have become more and more socially accept in Germany^23^. Thus, patients may have reported mental symptoms more openly, which may have been led to an increased coding by the medical practicioner. Compared to findings in the literature, the percentage of patients with psychological comorbidities was lower. For instance, in a cross-sectional survey of 1,021 German OA patients with a mean age of 66 years, 19.8% of male and 19.2% of female participants reported a score of ≥ 15 on the Patient Health Questionnaire-9 indicating a moderately severe depression. Predictors included perceived pain, few social contacts, physical limitation and body mass index^24^. Also, Sale and colleagues conducted a cross-sectional study in Canada, reporting that 21.3% of 1227 OA patients with a mean age of 75 years were classified as depressed using the Center for Epidemiologic Studies Depression Scale^25^. Narazinasab et al. found that 58.5% of OA patients in Iran had mental health disorders (n = 94, 68% female, mean age 44 years). They further showed the risk of mental health disorders was greater in the first months of OA diagnosis compared to patients with a disease duration of more than 6 months (*p* = 0.01). Additionally, the risk was significantly elevated for patients taking corticosteroids^26^. A recent meta-analysis included 49 studies reporting a pooled prevalence of depressive symptoms in 15,855 OA patients (59% female, mean age 65 years) of 19.9% (95% confidence intervals (CI): 15.9–24.5%, n = 10,811). For anxiety symptoms, the pooled prevalence was estimated to be 21.3% (95% CI 15.5–28.5%; n = 1226). For both diagnoses the relative risk was higher among patients with OA (1.17 for depression, n = 941 and 1.35 for anxiety, n = 733)^27^.

Further, the results also showed a decrease in certain psychological comorbidites, most pronounced regarding F5, behavioural syndromes associated with psychological disturbances and physical factors in both cohorts. As this chapter captures, among other, eating disorders, the reduction in diagnosis frequency might be attributable to advanced efforts over the past years to unreveal the relationship between OA and nutrition^28^. In the same stance, nutritional interventions and supplementation have been more and more considered in clinical settings^29,30^.

In light of the presented findings, a routine psychological screening for OA patients is deemed as a beneficial future direction considering that concomitant diagnosis of the chapter F of the ICD-10 are associated with worse clinical outcomes. For instance, a systematic review revealed that OA patients diagnosed with anxiety and/or depression experienced more pain, had frequent hospital visits, took more medication, and reported less optimal outcomes^31^. Also, depression and anxiety were found to be predictors of complications after primary total joint arthroplasty and have been associated with higher healthcare costs and extended length of hospital stay^12,14,32^. Depression was further shown to be among risk factors increasing the 90-days postoperative mortality after TKA^33^. Besides, other studies found an association between OA and perceived memory loss^34^ as well as 1.27 times increase in the odds of suicidal ideation^35^.

Additionally, the raising numbers of psychological comorbidities over the years shown in this study advocate for the implementation of mental and psychological support in orthopedics and trauma surgery. Studies on management strategies reported first insights^31^. One study demonstrated that patient education programs resulted in no clinically significant benefit^36^. However, in an evaluation of such a patient education program, it was found that the group with the lowest knowledge about the condition experienced more pain, whereas the group with the highest knowledge showed better coping and less depression (p < 0.05)^37^. In line with this finding, showing video informations on pre-operative anxiety to patients diagnosed with knee OA resulted in lower levels of anxiety and increased the tolerability of knee lavage^38^. Also, it was observed that exercise interventions were more beneficial when performed in groups than self-administered at home, suggesting that psychological support may play a role as a resource in the OA treatment^37^. Finally, systematic depression management (antidepressant pharmacotherapy and/or problem-solving treatment) over 12 months was shown to be more effective than usual care in decreasing pain severity^39^. Hence, to improve the management and patients’ quality of life, interdisciplinary collaboration is warranted and a psychologists or psychiatrists should be a part of the treatment team^40^.

The study shows several limitations. First, the ICD-10 codes do not allow a differentiation regarding treatment strategies. Also, the data could not be provided for age- or sex-specific subgroups. Further, it was not possible to derive individual features of the patients and risk factors such as obesity, and other somatic comorbidities, whether patients received treatment for psychological disorders or whether patients received medication, such as corticosteroid, which might have influenced the mental health. In the same stance, it was not possible to retrieve the onset of psychological symptom and therefore, it remains unclear to what extent the comorbidities can be attributed to OA. Additionally, analyzing large registry data does not allow to apply confirmatory diagnosis criteria. Whereas correct diagnosing is assumable, a possible upcoding cannot be excluded.

In conclusion, increasing psychological comorbidities advocate for the implementation of screening tools, prevention strategies, interdisciplinary approaches and psychological support in the treatment of osteoarthritis.

## Data Availability

The datasets used and analysed during the current study are available from the corresponding author on reasonable request.
